# Directed Evolution of Therapeutic Antibodies Targeting Glycosylation in Cancer

**DOI:** 10.3390/cancers12102824

**Published:** 2020-09-30

**Authors:** Ron Amon, Ronit Rosenfeld, Shahar Perlmutter, Oliver C. Grant, Sharon Yehuda, Aliza Borenstein-Katz, Ron Alcalay, Tal Marshanski, Hai Yu, Ron Diskin, Robert J. Woods, Xi Chen, Vered Padler-Karavani

**Affiliations:** 1 Department of Cell Research and Immunology, The George S. Wise Faculty of Life Sciences, The Shmunis School of Biomedicine and Cancer Research, Tel Aviv University, Tel Aviv 69978, Israel; ronamon@mail.tau.ac.il (R.A.); shahar22@yahoo.com (S.P.); sharonyehuda@mail.tau.ac.il (S.Y.); Marshanski1@mail.tau.ac.il (T.M.); 2 Department of Biochemistry and Molecular Genetics, Israel Institute for Biological Research, Ness-Ziona 76100, Israel; ronitr@iibr.gov.il (R.R.); rona@iibr.gov.il (R.A.); 3 The Azrieli Faculty of Medicine, Bar Ilan University, Safed 1311502, Israel; 4 Complex Carbohydrate Research Center, University of Georgia, Athens, GA 30606, USA; olgrant@uga.edu (O.C.G.); rwoods@ccrc.uga.edu (R.J.W.); 5 Department of Structural Biology, Weizmann Institute of Science, Rehovot 76100, Israel; aliza.katz@weizmann.ac.il (A.B.-K.); ron.diskin@weizmann.ac.il (R.D.); 6 Department of Chemistry, University of California, Davis, CA 95616, USA; hyu@ucdavis.edu (H.Y.); xiichen@ucdavis.edu (X.C.)

**Keywords:** cancer, tumor, glycosylation, antibodies, carbohydrate, polymer, nanoparticle, nanoprint

## Abstract

**Simple Summary:**

We generated a platform for designing optimized functional therapeutic antibodies against cancer glycans. The target tumor-associated carbohydrate antigen is commonly expressed in colon and pancreatic cancers. We developed a system for selection of potent antibodies by yeast surface display against this carbohydrate antigen, then showed that elite clones have potent affinity, specificity, cancer cell binding, and therapeutic efficacy. These tools have broad utility for manipulating and engineering antibodies against carbohydrate antigens, and provide major innovative avenues of research in the field of cancer therapy and diagnostics.

**Abstract:**

Glycosylation patterns commonly change in cancer, resulting in expression of tumor-associated carbohydrate antigens (TACA). While promising, currently available anti-glycan antibodies are not useful for clinical cancer therapy. Here, we show that potent anti-glycan antibodies can be engineered to acquire cancer therapeutic efficacy. We designed yeast surface display to generate and select for therapeutic antibodies against the TACA SLe^a^ (CA19−9) in colon and pancreatic cancers. Elite clones showed increased affinity, better specificity, improved binding of human pancreatic and colon cancer cell lines, and increased complement-dependent therapeutic efficacy. Molecular modeling explained the structural basis for improved antibody functionality at the molecular level. These new tools of directed molecular evolution and selection for effective anti-glycan antibodies, provide insights into the mechanisms of cancer therapy targeting glycosylation, and provide major methodological advances that are likely to open up innovative avenues of research in the field of cancer theranostics.

## 1. Introduction

Cancer is a leading cause of death worldwide and selective targeting by therapeutic monoclonal antibodies (mAbs) shows increasing success in modern oncology, mostly targeting proteins [1,2,3,4]. Cell surface glycosylation expression pattern is altered on cancer cells, leading to abnormal tumor-associated carbohydrate antigens (TACA) that are selectively and abundantly expressed on cancer cells [5,6]. These potentially valuable cancer cell surface targets are poorly immunogenic, hindering functional TACA-based cancer vaccines or immunotherapies, thus far [7,8,9]. Anti-carbohydrate antibodies typically have much lower affinities than antibodies recognizing proteins or peptide antigens (by 3–5 orders of magnitude), further complicated by carbohydrates large diversity in linkage types and modifications [10,11].

In 2018, The Nobel Prize in Chemistry was awarded to Frances H Arnold for inventing the directed evolution of enzymes, conjointly with George P. Smith and Sir Gregory P. Winter for their discoveries on phage display of peptides and antibodies. Yeast surface display (YSD) is one of the leading antibody engineering technologies to date, for both isolating novel antibodies and for directed evolution by *in vitro* affinity maturation of selected clones [12,13,14,15,16], allowing to identify mAb leads with good specificity and affinity. This system takes advantage of the agglutinin mating proteins (Aga1p and Aga2p) that are normally expressed on the yeast cell surface. These are expressed at 10^4^–10^5^ copies per cell, where Aga1p domain is anchored to the yeast cell wall and Aga2p is covalently attached to Aga1p through disulfide bonds [15,17]. In YSD, an antibody fragment is fused to the Aga2p allowing its cell surface presentation in accordance with the expression of the agglutinin proteins [18]. Most commonly, single chain fragment variable (scFv) or Fab antibody fragments are used in YSD, with surface expression and antigen binding monitored by flow cytometer allowing a very efficient sorting of large libraries according to antigen binding [18]. This system had mostly been employed against protein antigens [19,20].

Sialic acids (Sias) are acidic sugars found in vertebrates, topping cell surfaces glycans and glycoconjugates. Their expression patterns are altered on cancer cells [21,22,23], correlating with advanced stage, progression, and/or metastasis [24,25,26]. Thus, sialylated-TACA are promising targets for cancer therapy [27]. These include sialyl Lewis a (SLe^a^) found on pancreas, colorectal, stomach and liver cancers that suffer very short five-year survival rates [28,29]. The SLe^a^ tetrasaccharide Neu5Acα2−3Galβ1−3(Fucα1−4)GlcNAcβ1-R, namely CA19−9 antigen, is a cancer-associated marker widely used in clinical practice [30,31,32,33]. It is the only FDA-approved test for pancreatic cancer, but is also used in colorectal, gastric, or biliary cancers. It is utilized to monitor response to therapy; however, it is not useful for early detection, diagnosis, or therapy [33,34,35]. Different antibodies are used to measure CA19-9; however, there is great variability between measured outcomes [36,37]. Taken together, these findings suggest that antibodies of greater specificity and affinity against SLe^a^ carbohydrate antigen could potentially serve as better cancer theranostic tools. Importantly, such potent antibodies should recognize their glycan target in the right context as presented on cancer cells or shed from them. As a proof of concept for development of efficient cancer therapeutics targeting glycosylation, we cloned the most commonly used antibody against SLe^a^ (1116-NS-19-9) [38] into YSD platform that was used to obtain specific anti-SLe^a^ antibodies of high affinity and potency in cancer cells binding and cytotoxicity.

## 2. Results and Discussion

### 2.1. Generating YSD Library of 1116-NS-19-9 scFv

**Figure 1:**
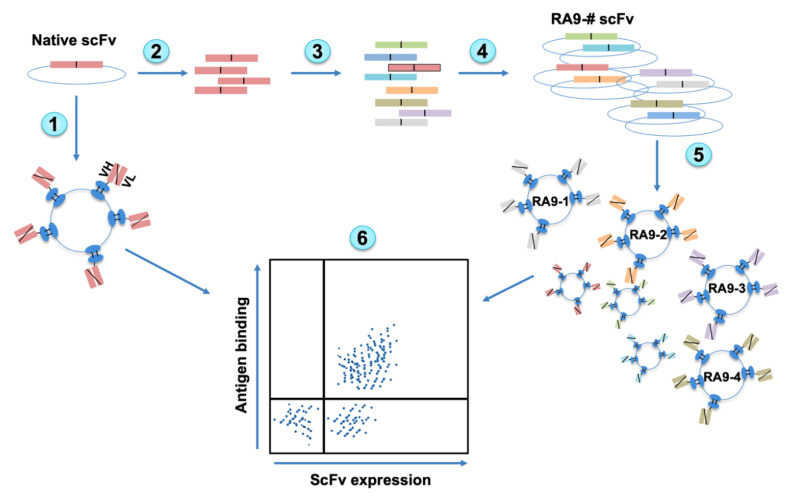
Overview of Yeast Surface Display (YSD) platform design. Native antibody scFv is cloned into YSD vector and yeast cells induced to express native scFv (1). The scFv fragment is PCR amplified (2) followed by error prone PCR to generate random mutagenesis (3). Mutated scFvs fragments are cloned into YSD vectors (4), transformed into yeast cells, and then induced to express scFv on cells surface (5). The scFv expression and antigen binding are measured by Fluorescence-Activated Cell Sorting (FACS) and top Ag^+^Ab^+^-double positive clones can be sorted out (6).

The sequences of the variable heavy chain (VH) and variable light chain (VL) fragments of 1116-NS-19-9 antibody were obtained from the ImMunoGeneTics (IMGT) database [39], and the native scFv with (G_4_S)_3_ linker between VH-VL was synthesized with flanking homology regions (RA9 native clone). To generate native-scFv expressing cells, competent yeast cells were transformed with *Nde*I/*Bam*HI digested pETCON2 plasmid and compatible synthetic-optimized scFv-DNA fragment. The resulting cloned scFv contained N’-HA tag and C’-c-Myc tag to facilitate evaluation of surface expression of the antibody fragment by Fluorescence-Activated Cell Sorting (FACS) after induction of transformed cells. Induced cells expressed surface native-RA9-scFv antibody fragment capable of binding the SLe^a^ antigen (Figure 1). YSD library of RA9-mutants-scFv was then generated by error prone PCR using the native scFv fragment as template, at low mutational loads (1–2 mutations/scFv). Mutated-fragments were cloned into the pETCON2 plasmid, then transformed into yeast cells representing 3.2 × 10^7^ different clones in the library. In the YSD system, enrichments can be achieved through multiple rounds of FACS sorting, allowing easy discrimination between clones with different affinities for the antigen, facilitating the isolation of higher affinity clones. Typically, 3 to 4 sorting cycles are required for the isolation of few superior clones against protein antigens [15,17]. Furthermore, the C’ tag also prevents accumulation of unwanted truncated clones during the selection process [15,17]. Mutated-RA9-scFv YSD library was analyzed and sorted against the glycan antigen with at least two-fold the number of cells than the library size (Figure 1).

A major characteristic of carbohydrates immune recognition is that it may be affected by glycans presentation mode. It was shown that glycan density, flexibility and polymer backbone rigidity can influence the functional affinity (avidity) of glycan binding proteins [40,41]. Thus, to fish out potent antibody clones we used a target antigen in a nanoparticle polymer expression mode resembling its presentation on cancer cells with multivalent expression on polyacrylamide polymers carrying biotin tags (7–9 glycans per ~30 kDa PAA-Bio; biotin at approximately every 5th amide group). Thus, we used nanoparticles multivalent-glycans in the form of polyvalent SLe^a^-PAA-biotin. We have not used monovalent glycans since they would not correctly mimic their expression on cancer cells. In addition, YSD selections are done in solution by FACS (rather than on a solid surface) better mimicking the natural presentation of carbohydrate antigens on cells [12,13].

**Figure 2:**
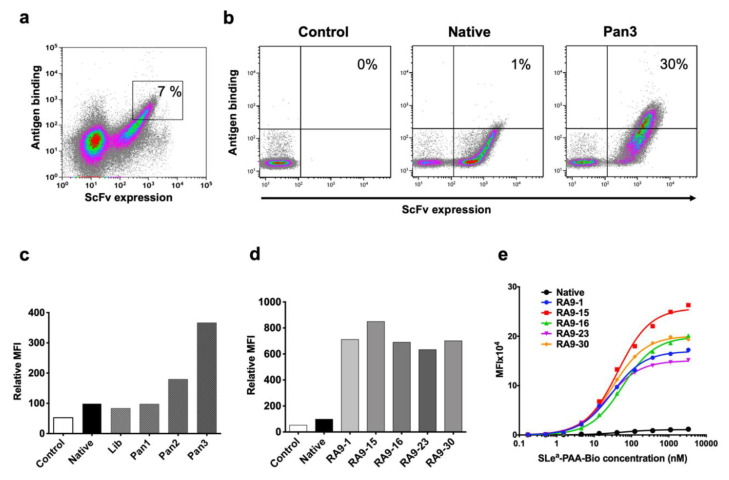
YSD library *in vitro* affinity maturation. (**a**) First cycle of FACS sort panning and selection. The top 7% clone-expressing yeast cells were sorted (marked), then further panned by two additional cycles. (**b**,**c**) Library panning results demonstrate improved binding (at 0.05 μM nanoparticle polymer SLe^a^-PAA-Biotin) with increased panning cycles. Unlabeled Pan3 negative control (Control) cells were stained only with secondary antibodies and compared to native YSD clone, mutated-RA9-scFv YSD library (Lib) and the three library panning cycles outputs (Pan 1, 2, 3). (**d**) Five single colonies from panning cycle 3 show high antigen binding (FACS against 0.05 μM SLe^a^-PAA-Biotin) compared to the native and unlabeled Pan3 control. (**e**) Apparent K_D_ was evaluated by FACS, binding of native and selected RA9-mutants clones expressed on yeast cells was examined at 10 serial dilutions of nanoparticle polymer SLe^a^-PAA-Biotin (3333–0.16 nM). In (**c**–**e**) cells were gated on single chain fragment variable (scFv) expressing cells and geometric mean fluorescence intensity of antigen binding measured. K_D_ was calculated according to non-linear fit with one-site specific binding using GraphPad Prism 8.0. Representative of two independent experiments.

Initial FACS sorting round was screened with 5 μM nanoparticle target (SLe^a^-PAA-Biotin) to accommodate the expected initial low affinity against this carbohydrate antigen. In addition, gates were drawn conservatively (7% of the library was collected; Figure 2a) to minimize mislay of improved clone. In subsequent sorts, conditions for sorting were used at increasing stringency by lowering nanoparticle antigen concentration and using highly-restricted gate sorting (second panning at 1 μM antigen, sorting top 1% binding cells; third panning at 0.1 μM antigen, sorting top 0.5% binding cells; Figure 2b,c). While the mutated-RA9-scFv YSD library initially showed reduced antigen binding compared to the native clone, after each panning sort there was a dramatic improvement in scFv expression (Figure 2b) and antigen binding by the library clones (Figure 2c). Yeast cells obtained by the third panning sort were cultured, then single colonies were collected and individually analyzed revealing a dramatic improvement in antigen binding compared to the native scFv clone (Figure 2d). Sequence analysis of selected mutated clones revealed that while only one scFv-clone contained a mutation in the framework region within the light chain variable fragment VL, more frequently mutations occurred in the framework and the Complementarity Determining Region (CDR) CDR1 regions within the heavy chain variable fragment VH, less common in CDR2, and none in the CDR3 region. These findings fit the common view that CDR3 has a pivotal role in antigen recognition, while changes in framework likely stabilize the antibody structure towards a more rigid state for optimal ligand fitting, eventually resulting in improved binding [42]. To further evaluate binding affinity, the apparent K_D_ values of scFv-yeast cells were measured by binding saturation curves against increasing nanoparticle antigen concentrations (ten serial dilutions ranging 3333–0.16 nM) revealing much higher affinities in each of the mutants compared to the native clone (Figure 2e). Altogether, these data provide compelling evidence for the feasibility of *in vitro* affinity maturation and directed evolution of antibodies against nanoparticle glycan antigens, at least in their scFv form.

### 2.2. Cloned Full Length Antibodies Maintain High Specificity and Affinity

**Figure 3:**
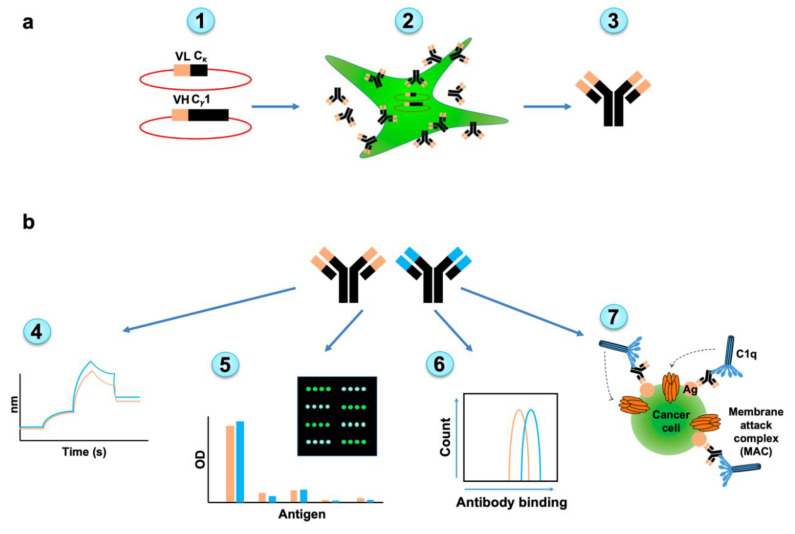
Overview of full-antibody expression and subsequent characterization. (**a**) Full length human IgG antibodies were generated from native and selected-mutants scFv clones. Variable heavy chain (VH) and variable light chain (VL) sequences were cloned into hIgG1/kappa vectors (1). The 293A cells were transfected to produce the full length antibodies (2). Antibodies were purified from cell media (3). (**b**) Full length antibodies were characterized for their affinity by bio-layer interferometry (BLI) (4), specificity by ELISA, and glycan microarray (5), cancer cell binding (6), and complement-dependent cellular cytotoxicity (7).

The evolved scFv fragments were next cloned and expressed as full-length IgG antibodies [43] for further characterization of their potency in terms of affinity, specificity, cancer cell binding and cytotoxicity (Figure 3). Gibson assembly was used to clone the VH and VL variable regions into corresponding p3BNC vectors that carry the constant regions (Figure S1). For each antibody chain (heavy and light), a separate vector containing a suitable constant region of hIgG1 or human kappa light chain was used. 293A cells (a sub clone of the human embryonic kidney cell line) were transfected with the plasmids and full-length antibodies purified by Protein-A through binding to antibody Fc region. Functional properties were then carefully analyzed by FACS, ELISA and by the powerful glycan microarray technology [44,45,46].

**Figure 4:**
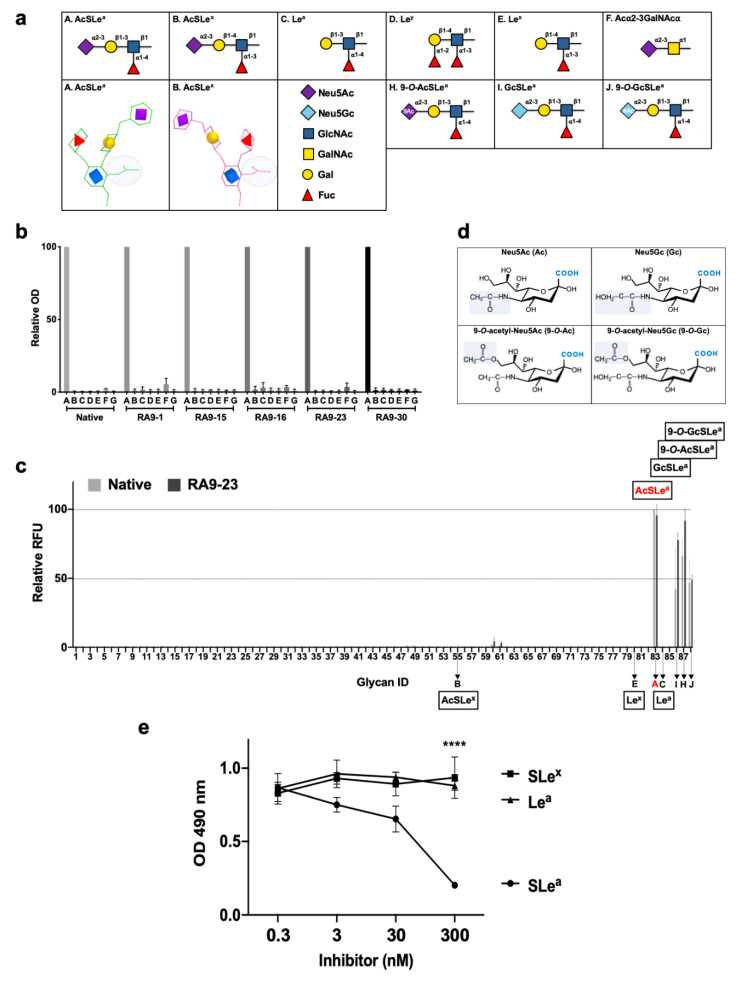
Cloned antibodies specificity against multiple glycan antigens. (**a**) Structures of SLe^a^ and closely-related glycans. In AcSLe^a^ (A), the *N*-acetyl group on GlcNAc (shaded grey circle) is oriented to the same side as the sialic acid (purple diamond), while in opposite directions in the AcSLe^x^ (B) isomer. (**b**) Specificity of native and RA9-mutated clones expressed as full-length IgG were examined by ELISA. 96-well plate was coated with PAA-polymer conjugated glycans, and primary antibodies examined at 10 ng/μL. Relative optical density (OD) was calculated as percentage of maximal binding of each antibody clone, followed by averaging the relative rank of two independent experiments (mean ± SEM). Glycans labeled A–F correspond to the structures described in (**a**), G is polyacrylamide backbone (PAA) control. (**c**) Binding of native and RA9-23 IgGs against diverse glycans was examined at 0.16, 0.08 and 0.032 ng/μL by a sialoglycan microarray (List of glycans in Table S1). Relative fluorescence units (RFU) was calculated as percentage of maximal binding at each concentration, followed by averaging the relative RFU rank of the three tested antibody concentrations for each glycan (mean ± SEM). A–C, E, H–J correspond to the structures described in (**a**). (**d**) Chemical structures of sialic acid types Neu5Ac (Ac), Neu5Gc (Gc), 9-*O*-acetyl-Neu5Ac (9-*O*-Ac), and 9-*O*-acetyl-Neu5Gc (9-*O*-Gc). (**e**) Specificity of the full-length antibody mutant clone RA9-23 was examined by ELISA inhibition assay against coated SLe^a^-PAA-Biotin, after pre-incubation of the antibody with specific (SLe^a^) or non-specific glycans (SLe^x^ and Le^a^). **** *p* < 0.001.

ELISA assay against five different nanoparticles of SLe^a^-closely-related PAA-glycans [SLe^a^, SLe^x^, Le^a^, Le^y^, Le^x^, and a shorter sialic acid-containing glycan (Neu5Acα2−3GalNAcα); Figure 4a] showed that all cloned antibodies specifically bind the SLe^a^ glyco-nanoparticle antigen maintaining recognition pattern of the native clone, despite the mutagenesis during YSD library design (Figure 4b). This analysis also revealed that sialic acid is imperative for antigen recognition, since binding to Le^a^ antigen that lacks the sialic acid was very low (less than 3.3%) in all antibodies. While SLe^a^ and SLe^x^ share the same tetrasaccharide building blocks, there was no cross-reactivity with SLe^x^ (less than 1.8%; Figure 4b). This could be explained by the differential spatial organization of these isomers, where in SLe^a^ the GlcNAc *N*-acetyl group and the sialic acid face the same side, but oppositely oriented in SLe^x^ (Figure 4a), generating a completely different 3D structure that could not be recognized by the antibody. This is a striking example of how glycan-linkages are important and critical in glycan diversity and complexity in nature. Subsequently, the K_D_ of full-length antibodies were determined by kinetic measurements using bio-layer interferometry (BLI) against the multivalent nanoparticle SLe^a^-PAA-Biotin, revealing that all mutant-clones have improved high affinity compared to the native antibody clone (Table 1).

**Figure 5:**
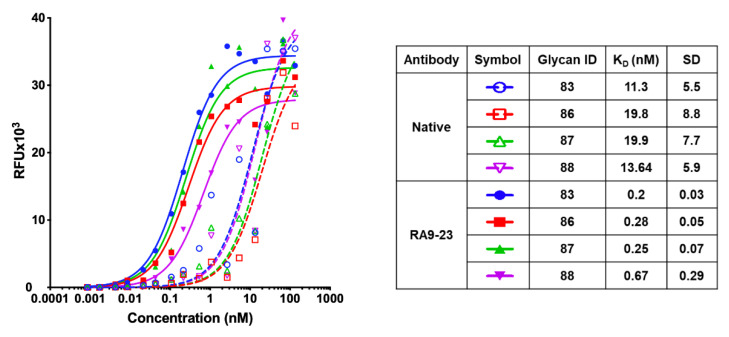
Apparent K_D_ of native and RA9-23 IgG by glycan microarray. Binding of native and RA9-23 IgGs was tested at 16 serial dilutions (133.3–0.000853 nM) against the top four bound glycans. Apparent K_D_ was calculated according to non-linear fit with one-site specific binding using GraphPad Prism 8.0.

We then used nanoprinted glycan microarrays to determine antibody specificity in a more detailed high-throughput assay against 88 different glycans, either non-sialylated or sialylated glycans that contain different types of sialic acids, including Neu5Ac (Ac), its hydroxylated form Neu5Gc (Gc) and their 9-*O*-acetylated derivatives (9-*O*-Ac/ 9-*O*-Gc) (Table S2). Mutant clone RA9-23, which showed top affinity by both scFv–YSD and full-length antibody, was analyzed. Array analysis showed that both the native and RA9-23 antibody clones are highly specific to AcSLe^a^ (glycan #83), GcSLe^a^ (glycan #86) and their corresponding 9-*O*-acetylated derivatives (glycan #87 and #88, respectively) (Figure 4c). The importance of the sialic acid and fucose residues for antibody recognition was also demonstrated, since Le^a^ (glycan #84) and Neu5Acα2−3Galβ1−3GlcNAcβProNH_2_ (non-fucosylated-SLe^a^, glycan #13) showed no binding at all. The specificity of this mutant clone was further demonstrated by ELISA inhibition assay, in which binding of RA9-23 to SLe^a^ was inhibited only with the specific glycan SLe^a^, but not with the closely-related glycans SLe^x^ or Le^a^ (Figure 4e). Further evaluation of affinities of these antibodies by saturation curves on glycan microarrays showed that RA9-23 antibody had more than 55-fold higher affinity than the native antibody against AcSLe^a^ (Figure 5). Even greater affinity improvement of >70-fold was measured against GcSLe^a^ (from 19.8 ± 8.8 to 0.28 ± 0.05) and 9-*O*-AcSLe^a^ (from 19.9 ± 7.7 to 0.25 ± 0.07). All of these antibody-recognized glycans (Figure 5) are tumor-associated carbohydrate antigens. SLe^a^ can either be populated with a terminal Neu5Ac or by Neu5Gc. Neu5Gc is a non-human sialic acid that can be consumed in the diet mostly from mammalian derived food [47], thereby replacing Neu5Ac and widely occurring in various cancer types [21,48]. Hence, GcSLe^a^ and 9-*O*-GcSLe^a^ are expected to appear more in cancer.

### 2.3. Molecular Modeling Demonstrates Stability of Mutant Antibody Clones

**Figure 6:**
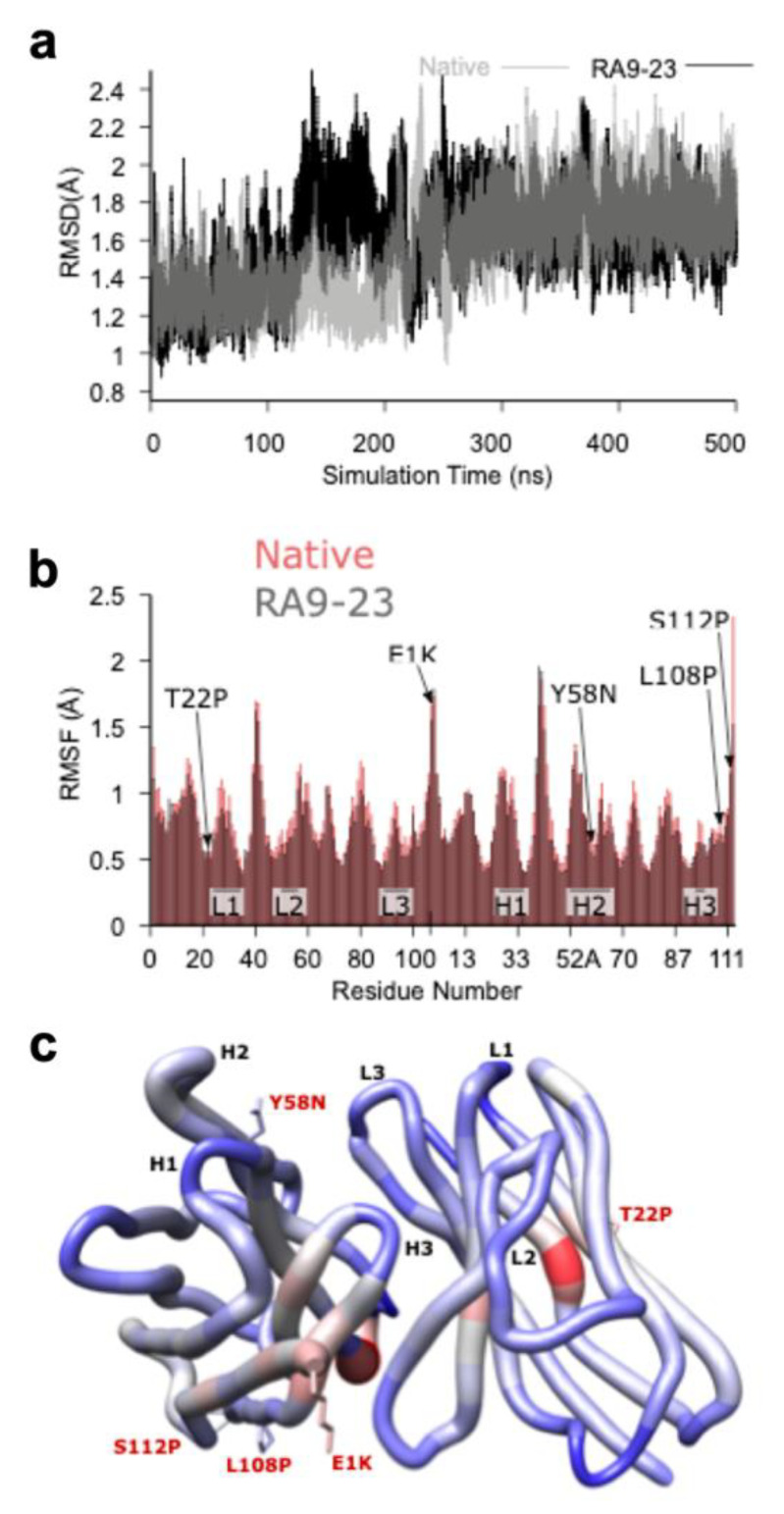
Molecular Modeling of the Fv Domain. (**a**) The root-mean-square deviation (RMSD) plot of the Cα atoms of both the native (grey) and RA9-23 mutant (black) clones structures over the course of the 500 ns molecular dynamics (MD) simulations, showing a structural rearrangement occurring between ~140 and 250 ns for RA9-23, and between ~200 and 250 ns for native. (**b**) A per-residue Cα atom root-mean-squared fluctuation (RMSF) plot for native (red) and RA9-23 (grey) clones. The plotted values are averages from five, 100 ns MD simulations that were started using 3D structures taken from the last 100 ns of the initial 500 ns simulation. The mutants and Complementarity Determining Regions (CDRs) are indicated. Most residues have lower RMSF values in the RA9-23 structure versus native (0.75 ± 0.05 versus 0.82 ± 0.03; *p* = 0.0277, *n* = 5, *t*-test). (**c**) A “worm” plot of the differences in Cα atom RMSF values between native and RA9-23 clones residues. The color range is from −0.3 (blue, more stable in RA9-23) to 0.0 (grey, no difference) to +0.3 (red, less stable in RA9-23). The sidechains are shown for residues that are mutated in RA9-23 versus native.

In order to gain further molecular insights into the mechanism of antibody-antigen binding, and to explain the improvement in mutated clone at the molecular level, homology models of the Fv domains of the native and RA9-23 were generated and subjected to 500 ns of molecular dynamics (MD) simulation. For both systems, some re-arrangement of the structures occurred during the 500 ns simulation (Figure 6a), but resulted in structures that were both similar to each other and stable for the remainder of the simulations. To further assess these new structures and allow for a statistical measure of differences between the two simulations, five other 100 ns simulations were started for both the native and RA9-23 systems using five structures taken at regular intervals from the last 100 ns of the initial 500 ns simulation. The average, per-residue root-mean-squared fluctuation (RMSF) for the Cα atoms was lower for RA9-23 versus the native clone (0.75 ± 0.05 versus 0.82 ± 0.03, *p* = 0.0277, *n* = 5, *t*-test), and agrees with the general observation that mutants that enhance affinity can do so by reducing backbone flexibility (Figure 6b). While some regions of the VH and VL chains were more flexible, in RA9-23 overall flexibility was reduced (Figure 6b,c).

### 2.4. Cloned Antibodies Are Effective at Cancer Cells Binding and Cytotoxicity

**Figure 7:**
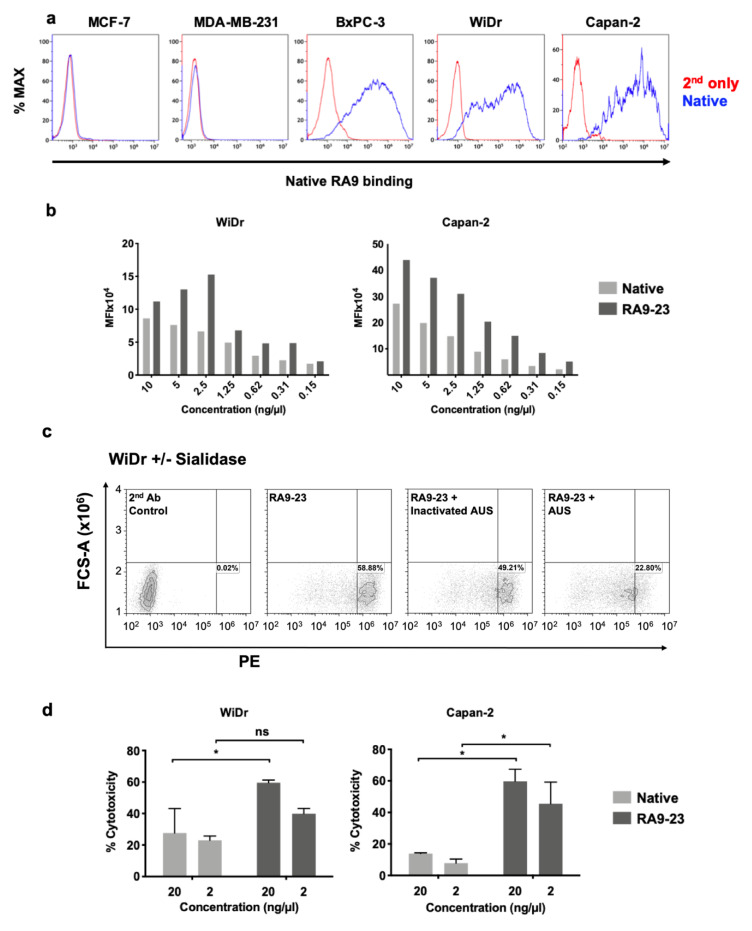
Antibody binding and cytotoxicity against cancer cells. (**a**) Binding of native antibody was examined by FACS against different cancer cell lines at 10 ng/μL (blue), compared to secondary antibody control (red). (**b**) Binding of native and RA9-23 IgGs to SLe^a^-expressing cancer cells (WiDr and Capan2) was examined by FACS at various concentrations (10–0.15 ng/μL). (**c**) Specificity of binding to cells was demonstrated by treatment of cells with *Arthrobacter Ureafaciens Sialidase* (AUS) that abrogated binding of RA9-23 IgG to SLe^a^-expressing WiDr cells, in comparison to direct binding of the antibody or its binding to cells treated with heat-inactivated AUS. (**d**) Complement-dependent cytotoxicity (CDC) of native and RA9-23 IgGs was examined. WiDr and Capan2 target cells were incubated with antibodies at concentrations 20 ng/μL and 2 ng/μL, then rabbit complement was added. Cytotoxicity was determined by LDH detection kit (representative of 2 independent experiments; 2-way ANOVA, * *p* < 0.05).

Cancer cell binding is critical for antibody therapeutic and diagnostic applications. Hence, cancer cell binding was examined to further evaluate antibody clones potency in the natural context. SLe^a^ expression was determined by native antibody and found positive in some cell lines (WiDr, Capan2, BxPC-3), while negative in others (MCF-7, MDA-MB-231) (Figure 7a). Thus, we followed up investigation on antigen-positive cells. We compared the binding of native and RA9-23 antibody clones to the SLe^a^-positive cancer cell lines WiDr and Capan2. RA9-23 showed higher binding efficiency compared to the native antibody clone in both cell lines, at various concentrations (Figure 7b), and with high specificity since binding was reduced dramatically after removal of sialic acids from the cell surface by a sialidase treatment (Figure 7c). Thus, RA9-23 antibody clone has high affinity against nanoparticle multivalent-glycans, and this is also reflected in the whole cell context, despite their heterogenous glycan expression patterns. It would be interesting to examine these novel antibody variants on patient samples in comparison to the native antibody. We next examined whether this improved cancer cell binding also translates into cancer cell killing. Antibodies of IgG1 isotype can facilitate cell killing by complement recruitment. Hence, complement-dependent cytotoxicity (CDC) was evaluated showing that RA9-23 clone has higher cytotoxicity in both WiDr and Capan2 cancer cell lines compared to the native clone (Figure 7d). These results exemplify the potential of this improved antibody clone for both detection of SLe^a^-positive tumors, and as cancer therapeutics.

## 3. Materials and Methods

### 3.1. Materials

Horseradish peroxidase (HRP)-goat-anti-human IgG (H+L), Cy3-goat-anti-human IgG (H+L) (Jackson ImmunoResearch), mouse-anti-c-Myc (clone 9E10) (Santa Cruz biotechnology), allophycocyanin (APC)-streptavidin (SouthernBiotech), Alexa-Fluor-488-goat-anti-mouse IgG1 (Life technologies). pETCON2 yeast surface display plasmid was kindly provided by Prof. Sarel Fleishman, Weizmann Institute of Science, Israel. Ovalbumin (Grade V, Sigma). We used nanoparticles polyvalent-glycans in the form of SLe^a^-PAA-Biotin, SLe^x^-PAA-Biotin, Le^a^-PAA-Biotin, Le^y^-PAA-Biotin, Le^x^-PAA-Biotin, Neu5Acα2−3GalNAcα-PAA-Biotin and PAA as control (GlycoTech).

### 3.2. Generation of YSD Library

Native sequences of 1116NS19.9 VH and VL were obtained from the IMGT database [39] (accession number S65761 and S65921, respectively). Native scFv of N’-VH and C’-VL with GGGGSGGGGSGGGGS linker was synthesized by Integrated DNA Technologies Inc. (IDT, Israel). The scFv DNA sequence was optimized for codon usage compatible with expression in human cells, without altering the amino acid sequence. In addition, the scFv sequence was flanked by plasmid homology regions at the 5′ and 3′ ends (36 and 45 nucleotides, respectively). The flanking regions contained 5′-*Nde*I and 3′-*Bam*HI restriction enzyme cloning site in-frame with the scFv.

pETCON2 plasmid contain HA and c-Myc tags to label the scFv. The N-terminal HA tag starts 30 amino acids upstream to VH, the C-terminal c-Myc tag starts 5 amino acids downstream to VL, and there is a GGGS linker in between the end of VL to the start of c-Myc tag. pETCON2 plasmid was digested with *Nde*I and *Bam*HI (Fermentas). Digested vector was extracted from 0.9% agarose gel using Wizard SV GEL & PCR clean-up system (Promega). EBY100 yeast cells were transformed with native scFv by LiAc/SS Carrier DNA/peg method, as described [49]. To generate RA9 library, scFv was first amplified by mixing 2 μL (20 ng) scFv template, 1 μL (10 μM) of each primer (Table S2a primers #1 and #2), 25 μL 2x ReddyMix PCR master mix (Thermo Scientific) and 21 μL PCR grade water. PCR settings were 95 °C for 2 min (min), followed by 30 cycles of 95 °C for 30 s (s), 55 °C for 30 s, 72 °C for 60 s and final incubation of 72 °C for 5 min. Amplified fragment was purified by Wizard SV Gel and PCR clean-up system then subjected to mutagenesis in two concentrations: either 100 ng template or 200 ng template. We then mixed 100 ng or 200 ng PCR products as template with 1 μL (10 μM) of each primer (Table S2a primers #1 and #2), 1 μL dNTPs (10 mM each dNTP), 5 μL buffer, 1 μL polymerase of GeneMorph II Random mutagenesis Kit (Agilent) complete to 50 μL with PCR grade water. Mutagenesis settings were 95 °C for 2 min followed by 17 cycles of 95 °C for 30 s, 60 °C for 30 s, 72 °C for 60 s and final incubation of 72 °C for 10 min. Products of mutated fragments (of the 100 ng and 200 ng original templates) were purified by Wizard SV GEL and PCR clean-up system then amplified by preparing five identical reaction mixes of 2 μL scFv template (5 tubes for 100 ng mutagenesis and 5 tubes for 200 ng mutagenesis), 1 μL (10 μM) of each primer (Table S2a primers #1 and #2), 25 μL 2× ReddyMix PCR master mix (Thermo Scientific), and 21 μL PCR grade water. PCR settings were 95 °C for 2 min followed by 30 cycles of 95 °C for 30 s 55 °C for 30 s, 72 °C for 60 s, and final incubation of 72 °C for 10 min. Amplified mutated fragments were purified from 2% agarose gel. To generate RA9 library, EBY100 yeast cells were prepared and electroporated as described [50]. Plasmids from native RA9 and mutated scFv sequence were analyzed at Tel Aviv University core facility (Table S2a primers #3 and #4).

### 3.3. Yeast FACS Staining and Sorting

RA9 yeast library was cultured in SD-Trp a synthetic defined media (SD) lacking Tryptophan (Trp) [2% glucose (Sigma), 0.67% yeast nitrogen base w/o amino acids (BD), 0.54% Na_2_HPO_4_ (Sigma), 0.86% NaH_2_PO_4_ (Sigma) and 0.192% yeast synthetic drop-out medium supplements without Trp (Sigma)] at 30 °C, passaged 1:10 each day for three days, then scFv was expressed by changing the media to SG-Trp a synthetic galactose (SG) based media [2% galactose (Sigma), 0.2% glucose, 0.67% yeast nitrogen base w/o amino acids, 0.54% Na_2_HPO_4_, 0.86% NaH_2_PO_4_, and 0.192% yeast synthetic drop-out medium supplements without Trp] and the temperature to 20 °C. For first panning 1 × 10^8^ yeast cells were washed with 1 mL assay buffer (PBS, 0.5% ovalbumin) then incubated with 5 μM SLe^a^-PAA-Biotin and 1:50 diluted mouse-anti-c-Myc, both in assay buffer for 1 h at room temperature (RT) with rotation. For the first panning cycle, cells were washed with 1 mL ice cold assay buffer, then incubated for 40 min on ice with APC-streptavidin and Alexa-Fluor-488-goat-anti-mouse IgG1 diluted 1:50 and 1:200 respectively in assay buffer. Cells were washed with 1 mL ice cold PBS, then top 5% of double positive (APC-Ag^+^AF488-Ab^+^) yeast cells were sorted into SD-Trp media using MoFlo Astrios EQ sorter (Beckman coulter). Recovered cells were cultured for few days in SD-Trp, then scFv was expressed by SG-Trp for a second panning. In this panning cycle 5 × 10^7^ cells were stained with 1 μM SLe^a^-PAA-Biotin (1/5 concentration of first panning cycle), while all other reagents were at the same concentration as in first panning. Top 1% of Ag^+^Ab^+^-double positive yeast were collected. In the third panning cycle, 1 × 10^7^ cells were stained with 0.1 μM SLe^a^ -PAA-Biotin (1/10 concentration of second panning cycle), while all other reagents were at the same concentration as in first panning. Top 0.5% of Ag^+^Ab^+^-double positive yeast were collected. Sorted cells were plated on SD-Trp agar plates and 30 single colonies were picked and cultured in SD-Trp.

### 3.4. Apparent K_D_ Calculations of Surface-scFv Expressed on Yeast Cells

scFv expressing yeast cells, of either native or RA9-mutant clones, were cultured in SD-Trp, then scFv expression inducted in SG-Trp media and stained for FACS analysis. The SLe^a^-PAA-Biotin antigen were added in ten serial dilutions ranging from 3333–0.16 nM in assay buffer (PBS + 0.5% ovalbumin). scFv expressing cells were gated and the geometric mean of antigen binding calculated. Geometric mean was plotted vs. antigen concentration and apparent K_D_ was calculated according to non-linear fit with one-site specific binding using GraphPad Prism 8.0.

### 3.5. Gibson Assembly

Plasmids of selected yeast clones were purified using Zymoprep Yeast Plasmid Miniprep II (Zymo Research) according to manufacturer’s instructions. Variable heavy and light fragments of native and selected clones were amplified by PCR. Reaction was made in Q5 reaction buffer, with 1 μL of plasmid DNA template (65–98 ng), 200 μM each dNTP, 1 U Q5 hot start high fidelity DNA polymerase (New England Biolabs), 500 nM each primer (Table S2b primers #5–10) complete volume to 50 μL with PCR grade water. PCR conditions were 95 °C for 2 min followed by 30 cycles of 95 °C for 30 s, 61 °C for 60 s, 72 °C for 60 s, and final incubation of 72 °C for 5 min. To remove template segments, the PCR product was supplemented with 6 μL of 10× CutSmart Buffer, 20 U DpnI (New England Biolabs), and completed the volume to 60 μL with PCR grade water, then incubated at 37 °C for 1 h. PCR digested fragments were purified from agarose gel by Zymoclean Gel DNA Recovery Kit (Zymo Research). Heavy and light chain full IgG p3BNC expression plasmids were divided to three parts for PCR amplification, variable region, left and right arms (Figure S1). Left and right arms of heavy and light p3BNC plasmids were amplified, digested and purified as described for variable regions using primers #11–16 (Table S2b). Of each fragment, variable region, right and left arms, 25 ng were taken for Gibson assembly. Reaction was made in isothermal reaction buffer containing 5% PEG 8000, 100 mM Tris-HCl pH 7.5, 10 mM MgCl_2_, 10 mM DTT, 0.2 mM of each dNTP and 10 mM NAD. To this buffer we added 0.04 U T5 exonuclease (NEB), 0.25U Phusion polymerase (NEB) and 40 U Taq DNA ligase (NEB), and ligation was made at 50 °C for 1 h. Plasmids were electroporated into XL1 *Escherichia coli*, to validate the sequence and producing high amount of p3BNC expression plasmids.

### 3.6. Expression and Purification of Full Antibody Clones

Human embryonic kidney 293A cells were used to produce whole native and RA9-mutant antibodies clones from the native and mutated RA9 p3BNC expression plasmids template transfected with polyethylenimine reagent (PEI; Polysciences), as described [51]. Antibodies were purified using protein A (GE healthcare) and concentrations determined by BCA assay (Pierce). For high antibody production levels, native and mutated RA9 p3BNC plasmids were transfected into HEK293F cells using PEI max transfection reagent (Polysciences), then after 6 days, medium sup was collected, centrifuged and filtered with addition of PMSF and azide. Medium was loaded on protein-A column (GE Life Sciences), eluted with 0.1 M of citric acid (pH 3.0) and brought to pH 7.0 with 2 M of Tris-HCl buffer (pH 8.0).

### 3.7. K_D_ Measurements of Full Antibody Clones

Polyvalent binding studies were carried out using the Octet Red system (FortéBio, Version 8.1, CA, USA) that measures bio-layer interferometry (BLI). All steps were performed at 30 °C with shaking at 1500 rpm in a black 96-well plate containing 200 μL solution in each well. Streptavidin-coated biosensors were loaded with 50 nM of biotinylated SLe^a^-PAA (or biotinylated Le^a^-PAA, as a negative control) for 300 s followed by washing step [with PBS buffer, pH 7.4, containing 10 mg/mL BSA and 0.1% (*v*/*v*) Tween 20]. Sensors were then reacted for 300 s with each antibody (native and selected clones) at increasing concentrations from 25 to 100 nM and then moved to buffer-containing wells for another 300 s (dissociation phase). Binding and dissociation were measured as changes over time in light interference after subtraction of parallel measurements from unloaded biosensors. Sensorgrams were fitted with a 1:1 binding model using the Octet data analysis software 8.1 (FortéBio).

### 3.8. Antibody Specificity by ELISA

Binding of antibodies to various glycans was tested by ELISA. PAA-glycans (GlycoTech) were coated in duplicates at 0.25 μg/well in 50 mM sodium carbonate-bicarbonate buffer, pH 9.5 onto 96-well microtiter plates (Costar, Corning) and plates were incubated overnight at 4 °C. Wells were blocked for 1 h at RT with blocking buffer (PBS pH 7.4, 1% ovalbumin). Blocking buffer was removed and primary antibody was added at 10 μg/mL in 100 μL/well in the same blocking buffer for two hours at room temperature. The plates were washed three times with PBST (PBS pH 7.4, 0.1% Tween) and subsequently incubated for 1 h at room temperature with HRP-goat-anti-human IgG 0.11 μg/mL in PBS. After washing three times with PBST, wells were developed with 140 μL of *O*-phenylenediamine in 100 mM citrate-PO_4_ buffer, pH 5.5, and the reaction stopped with 40 μL of H_2_SO_4_ (4 M). Absorbance was measured at 490 nm on SpectraMax M3 (Molecular Devices). Specific binding was defined by subtracting the background readings obtained with the secondary antibody only on wells coated with PAA. For ELISA inhibition assay, 96 well plate was coated with SLe^a^-PAA-Biotin (GlycoTech) in triplicates at 0.25 μg/well overnight at 4 °C. Wells were blocked with blocking buffer. RA9-23 antibody at 0.16 μg/mL was pre-incubated with either specific or non-specific target antigens (SLe^a^-PAA-Biotin and Le^a^-PAA-Biotin or SLe^x^-PAA-Biotin glycans, respectively) at 300–0.3 nM in blocking buffer. Antibody-glycan mixtures were incubated at 4 °C for two hours. Blocking buffer was removed from plate and antibody-glycan mixtures were added to the respective wells at 100 μL/well in triplicates, then incubated for two hours at room temperature, followed by washing, secondary antibody and substrate developing, as described above.

### 3.9. Cancer Cells Binding Assays

WiDr and Capan2 cells were obtained from American Type Culture collection (ATCC), MDA-MB-231 and MCF7 cells were a kind gift from Prof. Adit Ben-Baruch, BxPC3 cells were a kind gift from Prof. Ronit Satchi-Fainaro, both from Tel Aviv University. WiDr, Capan2, MDA-MB-231, and MCF7 cells were grown in Dulbecco’s Modified Eagle Medium (DMEM;biological industries) supplemented with 10% heat inactivated fetal bovine serum (FBS), 2 mM L-glutamine, 100 units/mL penicillin and 0.1 mg/mL streptomycin. BxPC3 cells were grown in Roswell Park Memorial Institute Medium (RPMI;biological industries) supplemented with 10% heat inactivated fetal bovine serum (FBS), 2 mM L-glutamine, 100 units/mL penicillin and 0.1 mg/mL streptomycin. For binding assays, cells were collected from plates using 10 mM EDTA. Moreover, 1 × 10^5^–3 × 10^5^ cells were incubated with native and RA9-23 antibodies diluted in FACS buffer (PBS with 0.5% fish gelatin) for 1 h on ice, followed by incubation with Cy3-AffiniPure goat-anti-human IgG diluted 1:100 in FACS buffer for 1 h on ice. Fluorescence was measured by CytoFLEX flow cytometry (Beckman Coulter). For sialidase FACS assay, WiDr cells were collected from plates using 10 mM EDTA. 0.5 × 10^6^ cells were divided into Eppendorf tubes and incubated for four hours at 37 °C with either PBS, 50 mU active *Arthrobacter Ureafaciens Sialidase* (AUS) (EY Laboratories, San Mateo, CA, USA) or 50 mU inactive AUS (pre-incubated in 90 °C for 30 min) in PBS. Then, cells were washed with FACS buffer, stained with 2.5 μg/mL RA9-23 antibody, followed by washing, secondary antibody labeling and fluorescence measurement, as described above.

### 3.10. Complement-Dependent Cytotoxicity (CDC) Assay

Rabbit complement (Sigma) was used. Cytotoxicity was evaluated by measuring lactate dehydrogenase (LDH) release using LDH Cytotoxicity Detection kit (Roche Applied Science) according to manufacturer’s instructions. All assays included maximum release control containing target cells with rabbit complement diluted 1:6 with 1% TritonX-100. For spontaneous background release control, cells were incubated only with rabbit complement. Percentage cytotoxicity was calculated as: ((test release–spontaneous release)/(maximum release–spontaneous release) × 100). 2 × 10^4^ target cells were incubated in triplicates with antibodies at 20 ng/μL and 2 ng/μL for 1 h on ice in 96-well round-bottom plates. Rabbit complement and triton diluted in RPMI were added and plates were incubated for 2 h at 37 °C. Then supernatants were collected and LDH release was determined.

### 3.11. Sialoglycan Microarray Nanoprinting

Arrays were fabricated with NanoPrint LM-60 Microarray Printer (Arrayit) on epoxide-derivatized slides (Corning 40044) with 16 sub-array blocks on each slide. Glycoconjugates were distributed into one 384-well source plates using 4 replicate wells per sample and 8 μL per well (Version 2.0). Each glycoconjugate (Table S1) was prepared at 100 μM in an optimized print buffer (300 mM phosphate buffer, pH 8.4). To monitor printing quality, replicate-wells of human IgG (80, 40, 20, 10, 5, 0.25 ng/μL in PBS + 10% glycerol) and AlexaFlour-555-Hydraside (Invitrogen A20501MP, at 1 ng/μL in 178 mM phosphate buffer, pH 5.5) were used for each printing run. The arrays were printed with four 946MP3 pins (5 μm tip, 0.25 μL sample channel, ~100 μm spot diameter; Arrayit). Each block (sub-array) has 20 spots/row, 20 columns with spot to spot spacing of 275 μm. The humidity level in the arraying chamber was maintained at about 70% during printing. Printed slides were left on arrayer deck over-night, allowing humidity to drop to ambient levels (40–45%). Next, slides were packed, vacuum-sealed and stored at RT until used.

### 3.12. Sialoglycan Microarray Binding Assay

Slides were developed and analyzed as previously described [44] with some modifications. Slides were rehydrated with dH_2_O and incubated for 30 min in a staining dish with 50 °C pre-warmed ethanolamine (0.05 M) in Tris-HCl (0.1 M, pH 9.0) to block the remaining reactive epoxy groups on the slide surface, then washed with 50 °C pre-warmed dH_2_O. Slides were centrifuged at 200× *g* for 5 min then fitted with ProPlate™ Multi-Array 16-well slide module (Invitrogen) to divide into the sub-arrays (blocks). Slides were washed with PBST (0.1% Tween 20), aspirated and blocked with 200 μL/sub-array of blocking buffer (PBS/OVA, 1% *w*/*v* ovalbumin, in PBS, pH 7.3) for 1 h at RT with gentle shaking. Next, the blocking solution was aspirated and 100 μL/block of purified antibodies in 20−1.28 × 10^−4^ ng/μL diluted in PBS/OVA were incubated with gentle shaking for 2 h at RT. Slides were washed three times with PBST, then with PBS for 2 min. Bound antibodies were detected by incubating with secondary detection diluted in PBS, 200 μL/block at RT for 1 h, Cy3-anti-human IgG 0.4 μg/mL (Jackson ImmunoResearch). Slides were washed three times with PBST then with PBS for 10 min followed by removal from ProPlate™ Multi-Array slide module and immediately dipping in a staining dish with dH_2_O for 10 min with shaking, then centrifuged at 200× *g* for 5 min. Dry slides immediately scanned.

### 3.13. Array Slide Processing and Apparent K_D_ Calculations

Processed slides were scanned and analyzed as described at 10 μm resolution with a GenePix 4000B microarray scanner (Molecular Devices) using 350 gain. Image analysis was carried out with GenePix Pro 6.0 analysis software (Molecular Devices). Spots were defined as circular features with a variable radius as determined by the GenePix scanning software. Local background subtraction was performed. Apparent K_D_ was calculated according to non-linear fit with one-site specific binding using GraphPad Prism 8.0.

### 3.14. Homology Modeling

A 3D structure of the Fv domain for both the native and RA9-23 mutant clone sequences was generated using the prediction of immunoglobulin structure (PIGS) web tool (http://circe.med.uniroma1.it/pigs) [52]. Structure templates with approximately 90% were found in the protein data bank (PDB) for both the VH (PDB ID = 1DLF) and VL chains (PDB ID = 3LIZ).

### 3.15. Molecular Dynamics Simulations of Homology Models

In silico modeling and molecular dynamics simulations can be used to compare between native and mutant antibodies, to better understand the molecular basis for antibody improvement in the mutant antibody. All simulations were performed using the Amber16 software suite. Using tleap [53], the 3D structures were placed in a cubic box of TIP5P [54] water with a 10 Å water buffer with counter ions added to neutralize the system. The FF14SB [55] force field with cut-offs of 10.0 Å for vDWS and 8.0 Å for electrostatics were employed. Initial energy minimization (10,000 steps steepest decent followed by 10,000 steps conjugate gradient) was performed with Cartesian restraints (5 kcal/mol throughout all phases) on all solute heavy atoms to optimize the water molecules position and orientation. The same restraints were employed during a 400 ps nPT equilibration phase at 300 °K. This was followed by a 1 ns structural equilibration phase with Cartesian restraints on protein Cα. The atom positions and velocities from the last step of equilibration were used to start a 500 ns production run, were no restraints were employed. Following an initial analysis, a further set of MD simulations were performed using five structures taken at regular intervals from the last 100 ns of the 500 ns production run using cpptraj [56]. Analyses of the trajectories were performed using cpptraj. Plots were generated using gnuplot. Figures with 3D structures were created using University of California San Francisco (UCSF) Chimera [57] or Visual Molecular Dynamics (VMD) [58].

### 3.16. Statistical Analysis

Statistical analysis conducted with Prism 8 with the specific methods as indicated in the figure legends.

## 4. Conclusions

Pancreatic and colorectal cancers are some of the most common cancers, yet these patients frequently suffer from late diagnosis [59]. Given the limited success of surgeries (15–20%), chemo and radiation therapies are the current standard of care [60]. The combination of late diagnosis with no efficient targeted therapy result in a very high mortality rate [59]. Discovery of new tools to target pancreatic and colorectal cancer cells might have a dramatic effect in the fight against these cancer types. Altered glycosylations in cancer provide potential targets for therapy [21,61,62], but despite numerous developed anti-glycan antibodies [63,64,65,66], most are not compatible with clinical applications. SLe^a^ is a potential therapeutic target in many types of cancers [28,29], including pancreatic and colorectal cancers [59,67,68]. Here, we demonstrate a platform for optimizing functional therapeutic antibodies against cancer glycans, exemplified by targeting SLe^a^.

## Figures and Tables

**Table 1:** K_D_ of selected RA9-mutant clones (RA9-mutant number #—as indicated), full-length antibody clones were measured by BLI against biotinylated SLe^a^-PAA or biotinylated Le^a^-PAA, as a negative control.

Antibody	K_on_ (1/Ms)	K_off_ (1/s)	K_D_ (nM)	K_D_ Native/ K_D_ RA9-mutant #
Native	5.4 × 10^4^	2.3 × 10^−3^	42	1
RA9-1	7 × 10^4^	6.3 × 10^−4^	9	4.7
RA9-15	1.2 × 10^4^	2.1 × 10^−3^	18	2.3
RA9-16	1.1 × 10^4^	1.2 × 10^−3^	11	3.8
RA9-23	1 × 10^4^	1.2 × 10^−3^	12	3.5
RA9-30	7.4 × 10^4^	2.7 × 10^−3^	37	1.1

## Supplementary Materials

The following are available online at https://www.mdpi.com/2072-6694/12/10/2824/s1, Figure S1: Gibson assembly of variable regions into p3BNC IgG expression vectors, Table S1: list of glycans nanoprinted on glycan microarrays, Table S2: list of primers.
