# Supplementary material for: Directed Evolution of Therapeutic Antibodies Targeting Glycosylation in Cancer
Source: Cancers (Basel). 2020 Sep 30;12(10):2824. doi: 10.3390/cancers12102824 (PMC7601599; doi:10.3390/cancers12102824)
Supplement: Supplementary file 1 [file cancers-12-02824-s001.pdf]

Supplementary Materials: Directed Evolution of Therapeutic Antibodies Targeting Glycosylation in Cancer

Ron Amon, Ronit Rosenfeld, Shahar Perlmutter, Oliver C. Grant, Sharon Yehuda, Aliza Borenstein-Katz, Ron Alcalay, Tal Marshanski, Hai Yu, Ron Diskin, Robert J. Woods, Xi Chen and Vered Padler-Karavani

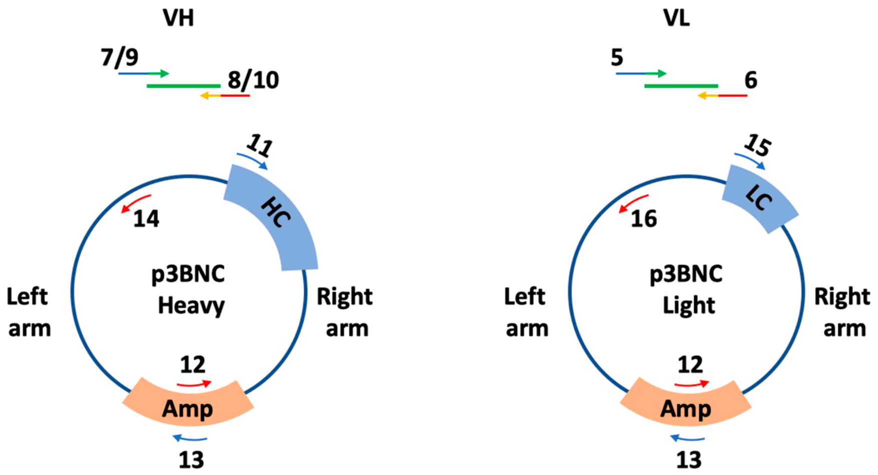

Figure S1. Gibson assembly of variable regions into p3BNC IgG expression vectors. Variable regions were cloned into p3BNC expression vectors by Gibson assembly. Primers number refer to the numbers in Supplementary Table 1b. HC-heavy constant, LC-light constant.

Table S1. List of glycans nanoprinted on glycan microarrays.

Glycan ID	Structure
1	Neu5,9Ac$_2$α3Galβ4GlcNAcβO(CH$_2$)$_2$CH$_2$NH$_2$
2	Neu5Gc9Acα3Galβ4GlcNAcβO(CH$_2$)$_2$CH$_2$NH$_2$
3	Neu5,9Ac$_2$α6Galβ4GlcNAcβO(CH$_2$)$_2$CH$_2$NH$_2$
4	Neu5Gc9Acα6Galβ4GlcNAcβO(CH$_2$)$_2$CH$_2$NH$_2$
5	Neu5Acα6GalNAcαO(CH$_2$)$_2$CH$_2$NH$_2$
6	Neu5Gcα6GalNAcαO(CH$_2$)$_2$CH$_2$NH$_2$
7	Neu5,9Ac$_2$α3Galβ3GlcNAcβO(CH$_2$)$_2$CH$_2$NH$_2$
8	Neu5Gc9Acα3Galβ3GlcNAcβO(CH$_2$)$_2$CH$_2$NH$_2$
9	Neu5,9Ac$_2$α3Galβ3GalNAcαO(CH$_2$)$_2$CH$_2$NH$_2$
10	Neu5Gc9Acα3Galβ3GalNAcαO(CH$_2$)$_2$CH$_2$NH$_2$
11	Neu5Acα3Galβ4GlcNAcβO(CH$_2$)$_2$CH$_2$NH$_2$
12	Neu5Gcα3Galβ4GlcNAcβO(CH$_2$)$_2$CH$_2$NH$_2$
13	Neu5Acα3Galβ3GlcNAcβO(CH$_2$)$_2$CH$_2$NH$_2$
14	Neu5Gcα3Galβ3GlcNAcβO(CH$_2$)$_2$CH$_2$NH$_2$
15	Neu5Acα3Galβ3GalNAcαO(CH$_2$)$_2$CH$_2$NH$_2$
16	Neu5Gcα3Galβ3GalNAcαO(CH$_2$)$_2$CH$_2$NH$_2$
17	Neu5Acα6Galβ4GlcNAcβO(CH$_2$)$_2$CH$_2$NH$_2$
18	Neu5Gcα6Galβ4GlcNAcβO(CH$_2$)$_2$CH$_2$NH$_2$
19	Neu5Acα6Galβ4GlcβO(CH$_2$)$_2$CH$_2$NH$_2$
20	Neu5Gcα6Galβ4GlcβO(CH$_2$)$_2$CH$_2$NH$_2$
21	Neu5Acα3Galβ4GlcβO(CH$_2$)$_2$CH$_2$NH$_2$
22	Neu5Gcα3Galβ4GlcβO(CH$_2$)$_2$CH$_2$NH$_2$
23	Neu5,9Ac$_2$α6GalNAcαO(CH$_2$)$_2$CH$_2$NH$_2$
24	Neu5Gc9Acα6GalNAcαO(CH$_2$)$_2$CH$_2$NH$_2$
25	Neu5Acα3GalβO(CH$_2$)$_2$CH$_2$NH$_2$

26	Neu5Gcα3GalβO$(CH_2)_2CH_2NH_2$
27	Neu5Acα6GalβO$(CH_2)_2CH_2NH_2$
28	Neu5Gcα6GalβO$(CH_2)_2CH_2NH_2$
29	Neu5,9Ac_2α3GalβO$(CH_2)_2CH_2NH_2$
30	Neu5Gc9Acα3GalβO$(CH_2)_2CH_2NH_2$
31	Neu5,9Ac_2α6GalβO$(CH_2)_2CH_2NH_2$
32	Neu5Gc9Acα6GalβO$(CH_2)_2CH_2NH_2$
33	Neu5Acα3Galβ3GalNAcβO$(CH_2)_2CH_2NH_2$
34	Neu5Gcα3Galβ3GalNAcβO$(CH_2)_2CH_2NH_2$
35	Neu5,9Ac_2α3Galβ3GalNAcβO$(CH_2)_2CH_2NH_2$
36	Neu5Gc9Acα3Galβ3GalNAcβO$(CH_2)_2CH_2NH_2$
37	Neu5,9Ac_2α6Galβ4GlcβO$(CH_2)_2CH_2NH_2$
38	Neu5Gc9Ac6Galβ4GlcβO$(CH_2)_2CH_2NH_2$
39	Neu5,9Ac_2α3Galβ4GlcβO$(CH_2)_2CH_2NH_2$
40	Neu5Gc9Ac3Galβ4GlcβO$(CH_2)_2CH_2NH_2$
41	Neu5Acα8Neu5Acα3Galβ4GlcβO$(CH_2)_2CH_2NH_2$
42	Neu5Acα8Neu5Acα8Neu5Acα3Galβ4GlcβO$(CH_2)_2CH_2NH_2$
43	Galβ4GlcβO$(CH_2)_2CH_2NH_2$
44	Galβ4GlcβNH_2
45	Galβ4GlcNAcβO$(CH_2)_2CH_2NH_2$
46	GalβNH_2
47	GalNAcαO$(CH_2)_2CH_2NH_2$
48	GalβO$(CH_2CH_2)_6NH_2$
49	GalβO$(CH_2)_2CH_2NH_2$
50	Galβ4GlcβO$(CH_2CH_2)_6NH_2$
51	Galβ3GalNAcβO$(CH_2)_2CH_2NH_2$
52	Galβ3GalNAcαO$(CH_2)_2CH_2NH_2$
53	Galβ3GlcNAcβO$(CH_2)_2CH_2NH_2$
54	Galβ4GlcNAc6SβO$(CH_2)_2CH_2NH_2$
55	Neu5Acα3Galβ4(Fucα3)GlcNAcβO$(CH_2)_2CH_2NH_2$
56	Neu5Gcα3Galβ4(Fucα3)GlcNAcβO$(CH_2)_2CH_2NH_2$
57	Neu5Acα3Galβ4(Fucα3)GlcNAc6SβO$(CH_2)_2CH_2NH_2$
58	Neu5Gcα3Galβ4(Fucα3)GlcNAc6SβO$(CH_2)_2CH_2NH_2$
59	Galβ3GlcNAcβ3Galβ4GlcβO$(CH_2)_2CH_2NH_2$
60	Neu5Acα3Galβ3GlcNAcβ3Galβ4GlcβO$(CH_2)_2CH_2NH_2$
61	Neu5Gcα3Galβ3GlcNAcβ3Galβ4GlcβO$(CH_2)_2CH_2NH_2$
62	Neu5Acα3Galβ4GlcNAc6SβO$(CH_2)_2CH_2NH_2$
63	Neu5Gcα3Galβ4GlcNAc6SβO$(CH_2)_2CH_2NH_2$
64	Neu5Acα8Neu5Acα3Galβ4GlcβO$(CH_2)_3NHCOCH_2(OCH_2CH_2)_6NH_2$
65	Neu5Acα8Neu5Acα8Neu5Acα3Galβ4GlcβO$(CH_2)_3NHCOCH_2(OCH_2CH_2)_6NH_2$
66	Neu5Acα6(Neu5Acα3)Galβ4GlcβO$(CH_2)_2CH_2NH_2$
67	Neu5Acα6(Neu5Gcα3)Galβ4GlcβO$(CH_2)_2CH_2NH_2$
68	Neu5Acα6(Kdnα3)Galβ4GlcβO$(CH_2)_2CH_2NH_2$
69	Neu5Gcα8Neu5Acα3Galβ4GlcβO$(CH_2)_2CH_2NH_2$
70	Kdnα8Neu5Acα3Galβ4GlcβO$(CH_2)_2CH_2NH_2$
71	Neu5Acα8Kdnα6Galβ4GlcβO$(CH_2)_2CH_2NH_2$
72	Neu5Acα8Neu5Gcα3Galβ4GlcβO$(CH_2)_2CH_2NH_2$
73	Neu5Acα8Neu5Gcα6Galβ4GlcβO$(CH_2)_2CH_2NH_2$
74	KDNα8Neu5Gcα3Galβ4GlcβO$(CH_2)_2CH_2NH_2$
75	Neu5Gcα8Neu5Gc-α3Galβ4GlcβO$(CH_2)_2CH_2NH_2$
76	Neu5Acα8Neu5Acα6Galβ4GlcβO$(CH_2)_2CH_2NH_2$
77	Neu5GcMeα8Neu5Acα3Galβ4GlcβO$(CH_2)_2CH_2NH_2$
78	Galα3Galβ4GlcNAcβO$(CH_2)_2CH_2NH_2$
79	Galβ3GalNAcαO$(CH_2)_2CH_2NH_2$
80	Galβ4(Fucα3)GlcNAcβO$(CH_2)_2CH_2NH_2$
81	Neu5Acα8Neu5Acα3Galβ4GlcO$(CH_2)_2CH_2NH_2$

82	Neu5Acα8Neu5Acα3(GalNAcβ4)Galβ4GlcO$(CH_2)_2CH_2NH_2$
83	Neu5Acα3Galβ3(Fucα4)GlcNAcβO$(CH_2)_2CH_2NH_2$
84	Galβ3(Fucα4)GlcNAcβO$(CH_2)_2CH_2NH_2$
85	Fucα2Galβ3(Fucα4)GlcNAcβO$(CH_2)_2CH_2NH_2$
86	Neu5Gcα3Galβ3(Fucα4)GlcNAcβO$(CH_2)_2CH_2NH_2$
87	Neu5,9Ac2α3Galβ3(Fucα4)GlcNAcβO$(CH_2)_2CH_2NH_2$
88	Neu9Ac5Gcα3Galβ3(Fucα4)GlcNAcβO$(CH_2)_2CH_2NH_2$

Table S2. (**a**) Primers used for cloning and sequencing in YSD system. (**b**) Primers used for cloning and sequencing of variable regions from scFv into p3BNC vectors.

**a. Primers used in YSD system**		
**Primer #**	**Primer name**	**Primer sequence 5→3**
1	pETCON2 universal For	TCTGGTGGAGGCGGTAGCGGAGGCGGAGGGTCGGC
2	pETCON2 universal Rev	TTACAAGTCCTCTTCAGAAATAAGCTTTTGTTCGGATCCGCCCCC
3	pCON2-seq-For_primer	GTTCCAGACTACGCTCTGCAGG
4	pCON2-seq-Rev_primer	GATTTTGTTACATCTACACTGTTG

**b. Primers for whole IgG exprssion**		
**Primer #**	**Primer name**	**Primer sequence 5→3**
1	p3BNC heavy for seq	GGATTCCCCGTGCCAAG
2	p3BNC heavy rev seq	CGGGAGATCATGAGGGTG
3	p3BNC light for seq	CTTCGTTAGAACGCGGC
4	p3BNC light rev seq	TCCTTGCTGTCCTGCTC
5	Native VL p3BNC for	GTAGCAACTGCAACCGGTGTACATTCAGACATTAAAATGACTCA GAGTCCATC
6	Native VL p3BNC rev	GGTGCAGCCACCGTACGTTTGATTTCTAATTTTGTTCCACCGC
7	Native VH p3BNC for	GTAGCAACTGCAACCGGTGTACATTGCGAAGTCAAGCTAGAAGA AAGCG
8	Native VH p3BNC rev	GATGGGCCCTTGGTCGACGCGGCAGAAACCGTCACCAG
9	RA9-23 VH p3BNC for	GTAGCAACTGCAACCGGTGTACATTGCAAAGTCAAGCTAGAAGA AAGCG
10	RA9-23 VH p3BNC rev	GATGGGCCCTTGGTCGACGCGGCAGGAACCGTCACCGG
11	p3BNC_H vector right arm for primer	GCGTCGACCAAGGGCCCATC
12	p3BNC_H/L vector right arm rev primer	CAACCAAGTCATTCTGAGAATAG
13	p3BNC_H/L vector left arm for primer	CTATTCTCAGAATGACTTGGTTG
14	p3BNC_H vector left arm rev primer	GCAATGTACACCGGTTGCAG
15	p3BNC_L vector right arm for primer	CGTACGGTGGCTGCACCATC
16	p3BNC_L vector left arm rev primer	TGAATGTACACCGGTTGCAGTTG

© 2020 by the authors. Licensee MDPI, Basel, Switzerland. This article is an open access article distributed under the terms and conditions of the Creative Commons Attribution (CC BY) license (http://creativecommons.org/licenses/by/4.0/).
